# MiR-499 Responsive Lethal Construct for Removal of Human Embryonic Stem Cells after Cardiac Differentiation

**DOI:** 10.1038/s41598-019-50899-2

**Published:** 2019-10-10

**Authors:** Edan Elovic, Sharon Etzion, Smadar Cohen

**Affiliations:** 1 The Avram and Stella Goldstein-Goren Department of Biotechnology Engineering, Ben-Gurion University of the Negev, Beer-Sheva, 84105 Israel; 2 Regenerative Medicine and Stem Cell (RMSC) Research Center, Ben-Gurion University of the Negev, Beer-Sheva, 84105 Israel; 3 The Ilse Katz Institute for Nanoscale Science and Technology, Ben-Gurion University of the Negev, Beer-Sheva, 84105 Israel

**Keywords:** Stem-cell biotechnology, Apoptosis, Embryonic stem cells

## Abstract

Deriving cell populations from human embryonic stem cells (hESCs) for cell-based therapy is considered a promising strategy to achieve functional cells, yet its translation to clinical practice depends on achieving fully defined differentiated cells. In this work, we generated a miRNA-responsive lethal mRNA construct that selectively induces rapid apoptosis in hESCs by expressing a mutant (S184del) Bax variant. Insertion of miR-499 target sites in the construct enabled to enrich hESC-derived cardiomyocytes (CMs) in culture. A deterministic non-linear model was developed and validated with experimental data, to predict the outcome for each treatment cycle and the number of treatment cycle repetitions required to achieve completely purified cTNT-positive cells. The enriched hESC-CMs displayed physiological sarcomere orientation, functional calcium handling and after transplantation into SCID-NOD mice did not form teratomas. The modular miRNA responsive lethal mRNA construct could be employed in additional directed differentiation protocols, by adjusting the miRNA to the specific cells of choice.

## Introduction

In recent years, human embryonic stem cells (hESCs) have emerged as an important source to generate large scale, lineage-committed progenitors capable of differentiating into functional cardiomyocytes^1^. Yet, the translation into the clinic of hESC-derived cardiomyocytes (hESC-CMs) has been challenged by the need to produce a fully defined differentiated cell population without the risk of tumorigenicity^2^. Directed cardiac differentiation protocols, which mimic mesoderm developmental signaling pathways by application of small molecules and/or growth factors, have been established and have undergone various modifications throughout the years^3–6^. Nevertheless, the differentiation efficiency is still often inconsistent in terms of hESC-CM yields, low reproducibility from batch to batch and incapability of generating uniform hESC-CMs^7^. While several purification strategies based on selective elimination of undifferentiated hESCs were reported^8–12^, only the metabolic selection method has been employed in practice^13–16^. By subjecting the cells to a glucose-depleted and lactate-enriched medium, this method exploits the ability of hESC-CMs to metabolize lactate as an alternative energy source to glucose. The lactate selection method enabled the enrichment of hESC-CMs and elimination of undifferentiated cells in late stage cultures. In early stage cultures, as the metabolic demands placed on the extensively proliferating cardiomyocytes are high, the metabolic selection method has been less efficient^10^. With this limitation, we aimed to develop a strategy capable of performing the enrichment in early-stage cultures of hESC-CMs. The recent study by Vunjak-Novakovic *et al*.^17^, showing that early–stage hPSC-CMs are more prone to undergoing maturation under mechanical stimulation, further supported the need for such method.

Our strategy takes advantage of the transient expression of certain microRNAs (miRNAs) during hESC differentiation^18^. MiRNAs function as negative regulators of gene expression at the translational level by silencing a targeted mRNA. Thereby, transition by cell-specific “miRNAomes” has the ability to distinguish and control transgene expression within hESCs and their derivatives. Recently, synthetic mRNA has emerged as a powerful tool^19^ and the use of sequence-engineered mRNA constructs has paved the way towards next generation miRNA-responsive exogenous protein expression in cells. A pioneering attempt was that of the Saito group, which incorporated miRNA targets in puromycin encoded modified mRNA for the efficient removal of undifferentiated hESCs after their prolonged exposure to antibiotics^20^.

In this work, we aim to enhance this approach by also taking into consideration the unique molecular mechanism of hESCs to undergo rapid apoptosis once DNA damage or DNA replication stress occurs^21,22^. This mechanism is highlighted by elevated activity of the pro-apoptotic Bax protein, which has been shown to be maintained in its activated conformation at the Golgi, ready to induce apoptosis once it is released to the cytoplasm^23^. While Bax in its native form is inactivated without stimulation, the mutant S184del variant has been shown to allow the protein to be in its activated conformation and induce apoptosis without any additional stimuli^24–26^. Therefore, we constructed a selective lethal mRNA that encodes for such a mutant Bax (mBax) in anticipation that after cardiomyocyte differentiation, it will selectively induce rapid apoptosis only in the remaining undifferentiated hESC population, while excluding hESC-CMs. To provide such selectivity, we inserted targets for the cardiac-abundant miR-499 along the lethal mRNA. Located in one of the introns of β-myosin heavy chain (*Myh7b*) gene, the expression of miR-499 is subjected to that of β-MHC^27^, leading to specific expression in cardiac and skeletal muscle cells alone, while it is essentially undetectable in hESCs^28^.

## Results

### A lethal mRNA construct expedites hESC death through apoptosis

A selective construct of miR-499-responsive lethal mRNA, encoding for the mutant S184del Bax (mBax_499 mRNA) was designed by inserting three repeats of miR-499 target in tandem along the 3′UTR with 4-mer gap. The construct was synthesized through *in vitro* transcription (IVT) using the viral T7 RNA polymerase, followed by purification and validation of the composition (Fig. S1.1).

**Figure 1:**
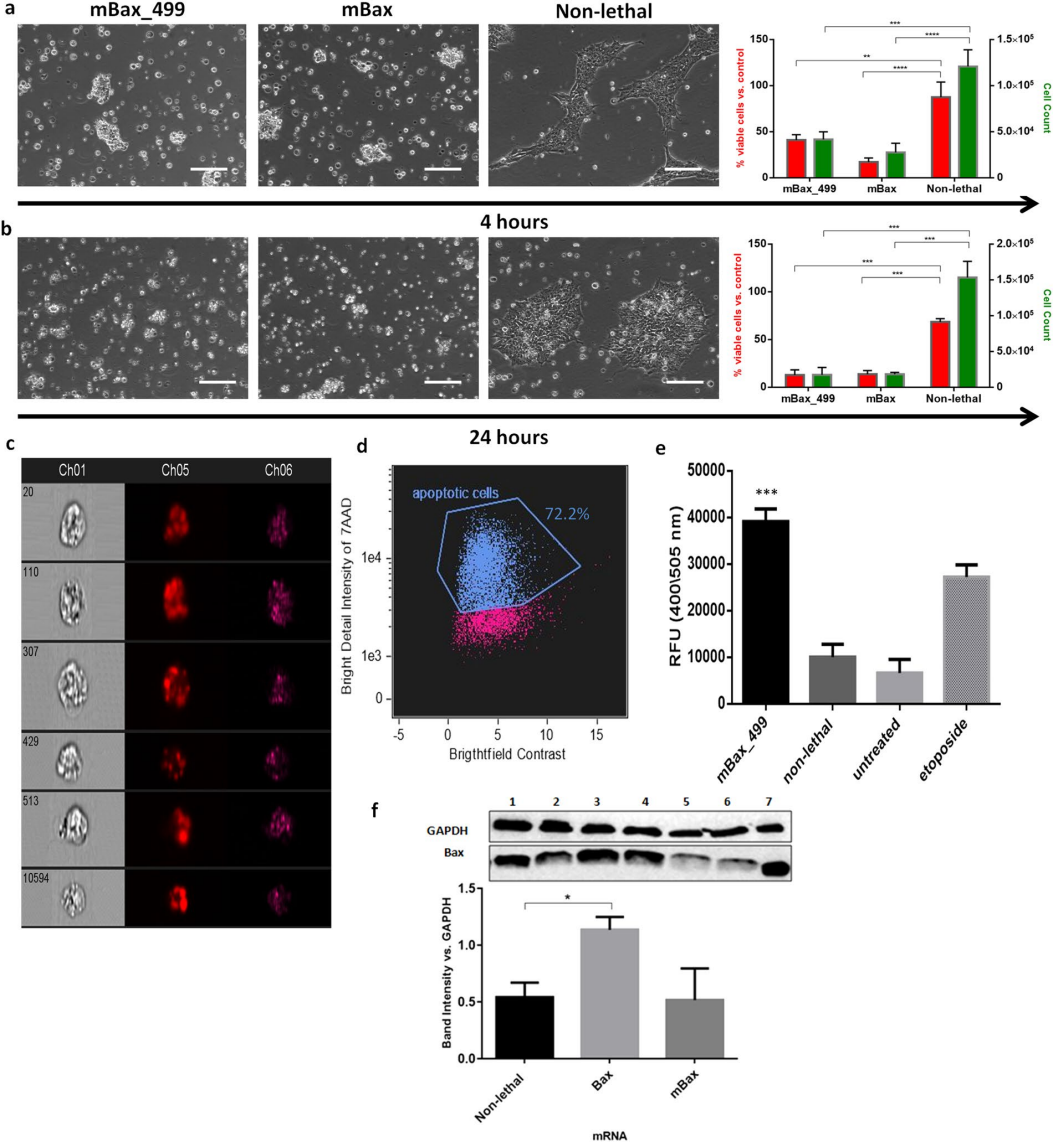
mBax_499 mRNA construct is lethal for hESCs. (**a,b**) Brightfield images of monolayer hESC cultures and measurement of cell viability and DNA content after (**a**) 4 h and (**b**) 24 h of treatment with various mRNAs. Scale bar: 130 µm. Cell viability (red) was evaluated by PrestoBlue assay and normalized to the value of control untreated cells (n = 3). Survival rates (green) were verified through DNA quantification by Hoechst 33342 after the removal of dead cells (n = 3). Values are according to calibration of intensity vs. cell count. (**c,d**) Observation of DNA fragmentation in selected images of single cells stained with 7AAD taken from ImageStream^X^ (**c**) showing brightfield (CH01), fluorescence 480 nm/615 nm (CH05) and SSC (CH06) and its analysis (**d**) using bright detail intensity R7 feature of positively stained cells for quantification of apoptotic events. (**e**) Caspase 3 activity of hESCs post treatment with either mBax_499 mRNA, non-lethal mRNA or etoposide (n = 3). (**f**) Western blot analysis of Bax protein level in hESCs post treatment (with: lanes 1:2 – eGFP mRNA, lanes 3:4 – Bax mRNA, lanes 5:6 – mBax mRNA) and its densitometric analysis (n = 2). Band intensity was normalized to the GAPDH housekeeping protein band intensity. Lane 7 – mBax protein produced by cell-free translation of mBax mRNA. All bar graphs indicate mean (SD). Full-length uncropped blots with low exposure are presented in Supplementary Fig. S1.3. *p* values were generated using one-way ANOVA with Tukey’s post-hoc test for multiple comparisons. *p < 0.5, **p < 0.01, ***p < 0.001, ****p < 0.0001, ns = not statistically different.

The lethality of the designated mBax_499 mRNA construct and the dynamics of this process were tested in cultures of undifferentiated hESCs. HESCs cultured as monolayers on Matrigel were transfected with the mBax_499 mRNA at a concentration of 2 pg/cell and were monitored for 24 h. The cells underwent rapid cell death as they began to deform and detach from the surface within 4 h from the start of treatment (Fig 1a, S2 movie). A similar behavior of cell death was observed in cultures treated with the mBax construct lacking the target for the cardiac-abundant miR-499 (mBax mRNA), indicating that the insertion of this sequence into the construct has no interference effect on mBAX expression (Fig. 1a). The lethal effect was a specific consequence of the mBax-encoding construct treatment, since control constructs, encoding for eGFP (non-lethal) or the nonactivated wild-type Bax, did not have such an effect on the cell colonies, which maintained the same appearance as untreated cells, i.e., remained attached to the surface and proliferated in the colonies (Fig. S1.2a). Moreover, comparison of cell viability following 24 h treatment with either mBax or Bax mRNA highlights the enhanced lethality of the mutant variant, reducing viability by 4-fold compared to the wild-type (Fig. S1.2b).

Quantification of the cell survival rates using Presto Blue assay for measuring cell metabolic activity (Fig. 1a, red bars) showed that within 4 h after treatment with the mBax constructs, there is a 60% reduction in cell metabolic activity compared to cultures treated with non-lethal constructs, indicating cell dismissal due to the mBax construct treatment. The result was supported by a similar reduction in cell count in the hESC cultures, as measured by Hoechst assay for DNA content (Fig. 1a, blue bars), compared to cell count in samples treated with non-lethal mRNA (see methods, Fig. 1a). By 24 h after transfection, over 85% of the cells treated with mBax constructs did not survive the treatment according to viability and cell count assays (Fig. 1b). The extensive cell death following treatment with the mBax construct was further confirmed by staining the cell cultures with 7-Aminoactinomycin D (7-AAD), which binds DNA in nonviable cells. Flow cytometry analysis after staining revealed that 95% of the cells were 7-AAD positive after 24 h of treatment with mBax_499 mRNA, indicating their death (Fig. S1.2c).

To substantiate that the mechanism of cell death is via apoptosis inflicted by the mBax constructs, we analyzed DNA fragmentation, which occurs during apoptosis. Single cell imaging of cells stained with 7-AAD provided indication of DNA fragmentation as multiple bright spots were identified inside the cells in various images (Fig. 1c). Image processing using bright detail analysis, which computes the intensity of the localized bright spots, was performed in order to quantify the percentage of DNA-fragmented cells and showed that the majority (72.2%) of the positively stained population underwent apoptosis (Fig. 1d).

Additionally, we measured Caspase 3 activity in the treated cells as this enzyme is activated during apoptosis. As a positive control group, the cells were treated with a known apoptosis inducer, Etoposide. By this assay, 24 h after treatment with mBax mRNA construct, Caspase 3 activity was 4-fold greater in the treated hESCs compared to its levels in hESCs treated with non-lethal mRNA construct or in untreated cells (Fig. 1e).

The efficacy of mBax to cause fatality in hESC cultures was further proven by following its translation to protein. Western Blot analysis was performed using an anti-Bax N-terminal (1–100 aa) antibody, which detects both the wild-type and the mutant variant (S184del) of Bax protein. This was confirmed by the identification of the cell-free translated mBax protein on the blot showing that the antibody detects mBax as well (Fig. 1f, lane 7). Unexpectedly, hESC extracts after 24 h of treatment with mBax mRNA construct revealed no measurable change in the Bax protein level compared to its level in hESCs treated with non-lethal mRNA, while the treatment with wild-type Bax mRNA resulted in an increased translation level of Bax (Fig. 1f, lanes 1–6). We interpret these results as evidence for the extreme ability of mBAX to cause rapid and efficient apoptosis; upon translation of even a minimal amount of mBax, it induces rapid and efficient cell death, before the cell produces a sufficient amount of the mutant mBax protein that can be detected in such an assay.

### MiRNA silencing mechanism suppresses apoptotic induction of miRNA-responsive mBax mRNA

**Figure 2:**
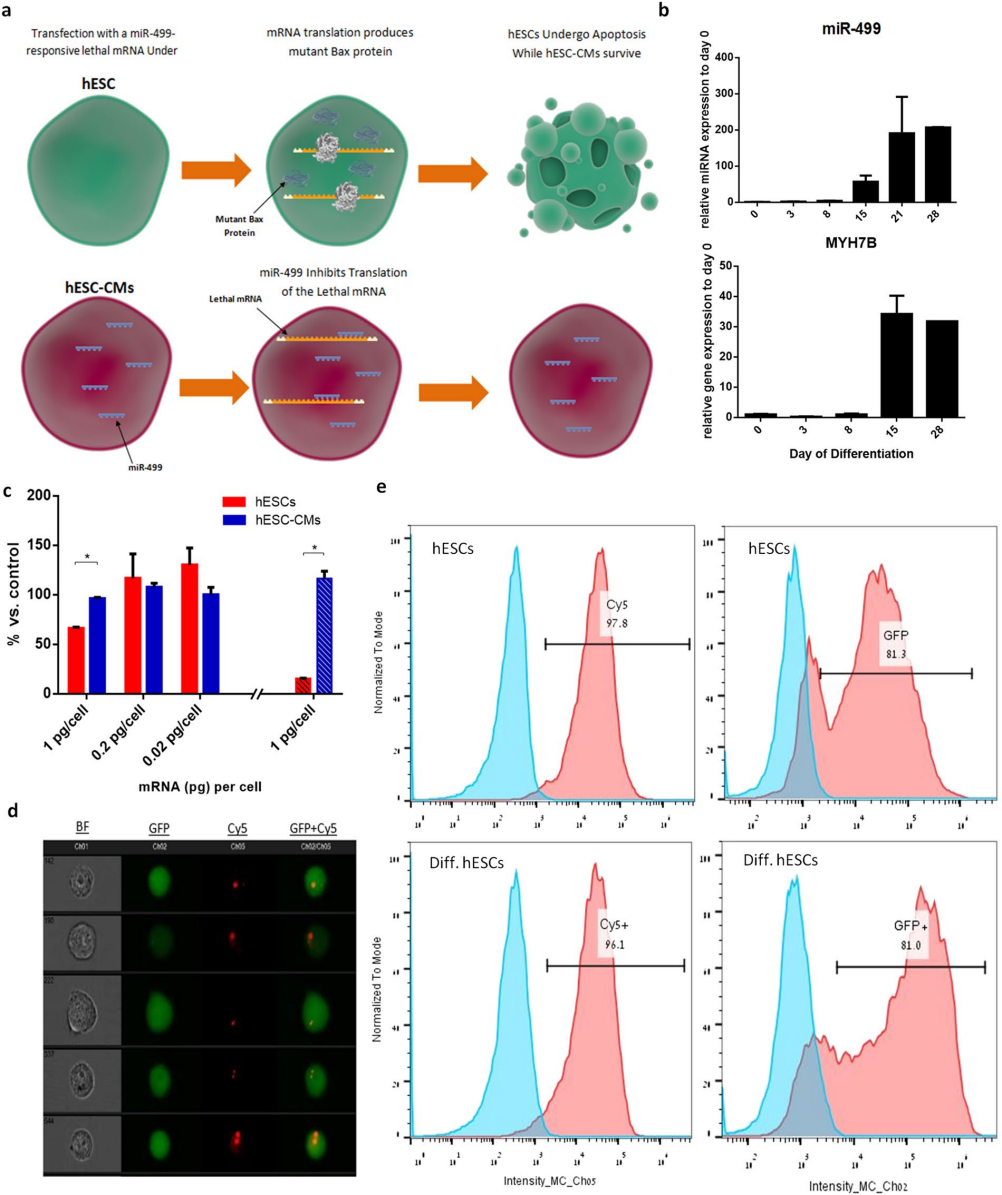
Optimization of mBax_499 construct for enrichment of hESC-CMs. (**a**) General scheme of possible outcome after treatment with mBax_499 mRNA. The scheme includes basic scientific illustrations downloaded from somersault1824.com. (**b**) Quantitative PCR analysis of miR-499 and its corresponding host gene MYH7B expression levels during directed cardiac differentiation. (**c**) hESC-CMs purified by FACS AriaII sorter after staining with SIRPa antibody were treated with various concentrations of mBax_499 mRNA construct. Presto Blue viability assay was performed for undifferentiated hESCs (red) and purified hESC-CMs (blue) after 2 h (plane bars) and 24 h (stripe bars) of treatment (n = 2). (**d,e**) Undifferentiated hESCs and cardiac differentiated hESCs were transfected with eGFP mRNA labelled with Cy5 using the Stemfect reagent. After 4 h of incubation, the cells were analyzed by Imagestream^X^. (**d)** Selected images of single cells presenting cellular uptake and localization of eGFP mRNA (red) and protein (green) captured by ImageStream^X^. (**e**) Analysis for Cy5 and GFP intensity distribution. Populations: blue - untreated cells, red - cells treated with eGFP mRNA labelled with Cy5. All bar graphs indicate mean (SD). *p* values were generated using two-tailed unpaired student’s *t-test*. *p < 0.05.

Due to the extreme lethality effect of mBax, utilizing the miRNA translation-regulator mechanism as an OFF switch of the construct demands tight inhibition of the exogenous mBax expression. In our designed construct, the OFF switch was miR-499 activity, which is highly expressed in cardiac differentiated cells and is absent in hESCs. We envisioned two possible scenarios to occur when applying the mBax_499 mRNA construct treatment (Fig. 2a). In one scenario (the top), treating the undifferentiated hESCs with the lethal construct in the absence of miR-499 in cells, translation of mBAX occurs, which kills the undifferentiated cells. In the second scenario involving cardiac differentiated hESCs (hESC-CMs), the expression of miR-499 in these cells and its binding to the target site on the mBax-499 prevents mBax translation, thus protecting the cells from the lethal effect of the mBax. To substantiate this outline, we examined whether the presence of miR-499 could efficiently function as the trigger of OFF switch in cells. Thus, hESCs were transfected with exogenous mature miR-499 mimic prior to treatment with mBax construct, with or without miR-499 targets sites. In hESC cells pre-treated with miR-499 mimic, the treatment with the nonselective mBax mRNA (without miR-499 target sites) construct led to cell death, while with the selective mBax_499 mRNA construct, cell survival was similar to cultures treated with nonlethal mRNA construct or not treated at all (Fig. S1.2d).

In an attempt to maximize the sensitivity of the mBax_499 mRNA construct towards miR-499, we investigated whether the insertion of an additional miRNA target site along the 5′UTR would increase the sensitivity of the OFF switch mechanism and consequently the cell protection from apoptosis inflicted by the treatment of mBax_499 construct. The additional miR-499 target site inserted in the 5′UTR of mBax_499 mRNA did not increase cell viability in comparison to treatment with the original mBax_499 mRNA, (Fig. S1.2d). Therefore, it was concluded that the insertion to the 5′UTR of mBax_499 mRNA has no additional effect.

We next tested the ability of the naturally expressed endogenous miR-499 in hESC-derived CMs to inhibit apoptosis induced by the mBax_499 mRNA construct in these cells. For this, we first analyzed the expression levels of miR-499 during cardiac differentiation in aim to select the appropriate time point for hESC-CMs enrichment with mBax_499 mRNA construct treatment. Highly dense monolayers of hESCs were subjected to cardiac cell differentiation via small molecule modulation of Wnt signaling^3^. By day 8–10 of differentiation, cardiac-related phenotypes started to appear, such as spontaneous contraction. RT-PCR analysis revealed that the transcript level of miR-499 was low up to day 8 of differentiation and from day 15 it was highly expressed (80-fold compared to day 0) and continued to increase with time, in correspondence with the elevation in the transcription level of its host gene, MYH7b (Fig. 2b). Therefore, we selected to test the effect of mBax_499 mRNA construct treatment on differentiated hESCs at day 15 of cardiac differentiation.

Sorted hESC-derived CMs by SIRPa surface marker did not show any reduction in cell viability during treatment with different concentrations of the mBax_499 mRNA, ranging from 0.02–1 pg/cell; they showed the same cell viability after 2 and 24 h (Fig. 2c, blue bars). By contrast, when treated with mBax_499 mRNA at a concentration of 1 pg/cell, the cell viability of undifferentiated hESCs decreased to less than 10% of its initial level (Fig. 2c, red bar). Treating hESCs with 0.2 pg/cell of mBax_499 mRNA or less did not affect cell viability, thus defining the 1 pg/cell concentration as the minimal lethal concentration (MLC) of the mBax_499 construct that will be used in the following assays (Fig. 2d).

To exclude the possibility that poor cellular uptake of the mBax_499 construct and its low translation rate are the reasons for the maintenance of hESC-CM survival, we compared the synthetic mRNA delivery rates in undifferentiated hESCs and their derivatives after cardiac differentiation. GFP encoding mRNA was labeled with the fluorescence label Cy5 (red) in order to evaluate both cellular uptake and cellular translation rates to form GFP (green). Imaging flow cytometer analysis revealed that by 4 h post transfection, cellular uptake of labeled mRNA had occurred in the vast majority of both undifferentiated hESCs and differentiated hESCs-CMs, 97.8% and 96.1%, respectively. Co-localization of the GFP expression (green) was observed in roughly 81.3% of both populations, suggesting that such synthetic mRNA has a translation efficiency of 81.3% (Fig. 2e,f) which apparently is not affected by differentiation, along with cellular uptake.

Taken together, our results show miR-499 to be an effective OFF switch of the lethal mBax construct, thus enabling the selective enrichment of hESC-derived CMs while eliminating undifferentiated hESCs.

### MBax_499 mRNA treatment enriches hESC-CMs in culture after 15 days of differentiation

**Figure 3:**
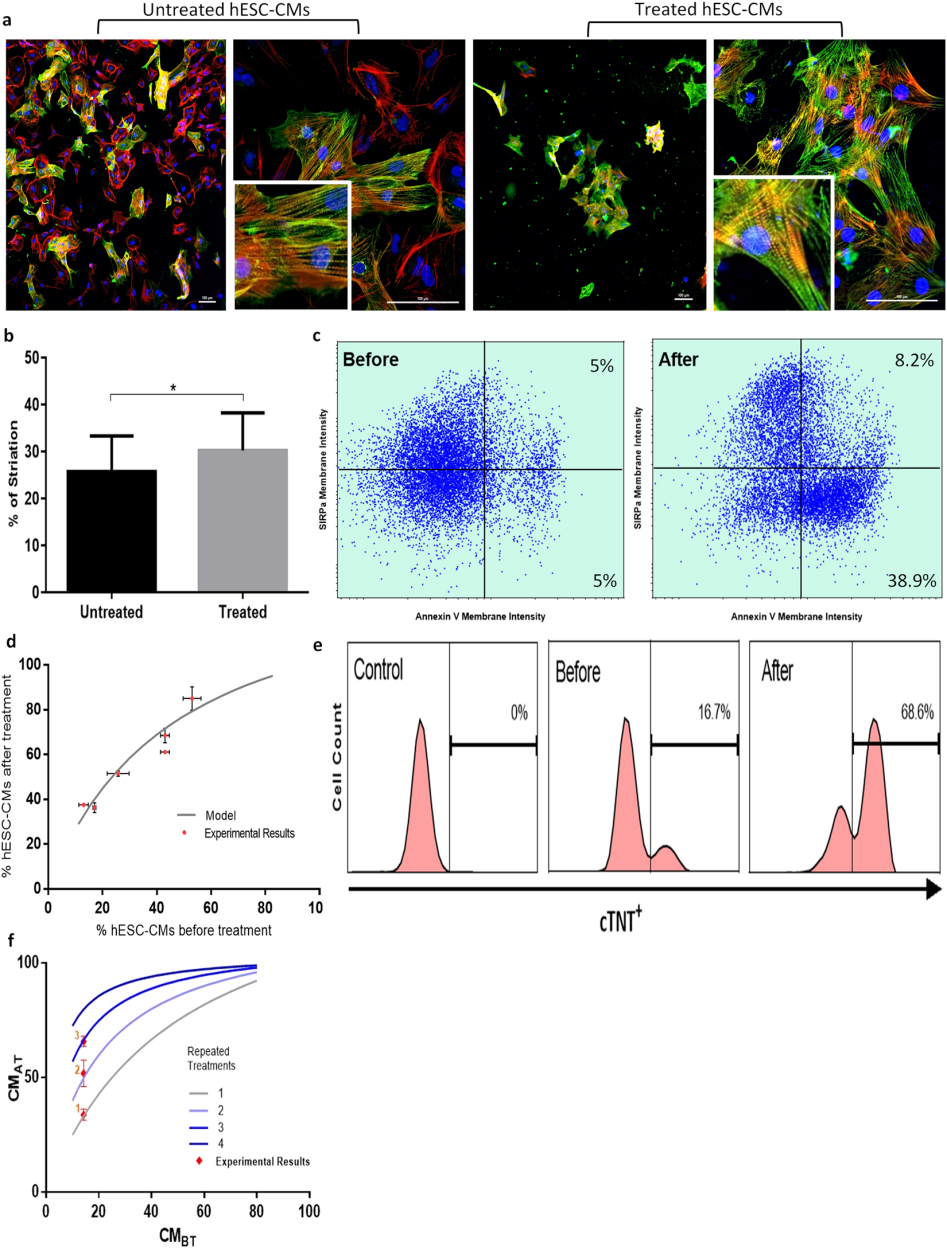
Enrichment of hESC-CMs by mBax_499 mRNA and modelling to predict yield. (**a**) Representative confocal images of hESC-CMs that were either untreated or treated with mBax_499 mRNA for 24 h and stained for F-actin filaments by phalloidin (red), counterstained with NucBlue (nucleus, blue) and sarcomeric α-actinin (green). Scale bar: 100 µm. (**b**) Quantification of the percentage of actinin fiber alignment along the major strain axis in randomly cropped confocal images of treated hESC-CMs (n = 27) and untreated hESC-CMs (n = 29) using the Image J software and the OrientationJ Distribution plugin. (**c**) Co-staining of cells with SIRPa-APC and Annexin V-FITC before treatment (left) and after 24 h treatment with mBax_499 mRNA (right). (**d**) Experimental results vs. model for outcome prediction of % hESC-CMs after treatment. (**e**) FACS analysis of cells stained with cTNT after three consecutive treatments with mBax_499 mRNA. (**f**) Utilizing the model for repetition of treatment prediction (one through four treatments) and comparison to experimental results of one, two and three repeated treatments with mBax_499 mRNA. Data are represented as mean (SD). *p* value was generated using two-tailed unpaired student’s *t-test*. *p < 0.05.

Next, we tested the capacity of the mBax_499 lethal mRNA construct in selectively removing any residual hESCs in culture and/or other sub-populations lacking miR-499 activity after cardiac differentiation and thus enriching the population with hESC-CMs. On day 15 of differentiation, we re-plated the cells on Matrigel at lower density (450,000 cell/well) in 12-well plates to enable follow-up of their appearance and morphology and the efficient delivery of the treatment construct. The cultures were then transfected with mBax_499 mRNA construct for 24 h, at a concentration of 1 pg/cell. Immunostaining of hESC-CMs prior and after enrichment with the mBax_499 constructs for sarcomeric α-actinin (green), filament F-actin (red) and nucleus (blue) and confocal imaging revealed the removal of nonmyocytes, those cells who were stained only red for F-actin but were negative to α-actinin. In the enriched cultures, visualization of random fields in the images showed no only-red stained cells; the cells were positively stained for both α-actinin and f-actin, denoting them as cardiomyocytes (Fig. 3a). Of note, the hESC-CMs in enriched cultures present a superior sarcomeric fiber morphology as reflected by the greater degree of cell striation (Fig. 3b) quantified by the Image J software and the Orientation J Distribution plugin (details in Supplementary Fig. S1.4). This result highlights the capability of mBax_499 mRNA construct treatment to enrich hESC-CMs after differentiation. The enrichment occurred via death and removal of undifferentiated cells as well as differentiating cells lacking miR-499 activity, without damaging the functionality and maturity of the cardiac differentiating cells.

To quantify the extent of enrichment, the percentage of cardiac troponin (cTNT) positive cells was measured before and after mBax_499 mRNA treatment in differentiated populations with varying percentages of hESC-CMs. In our hands, six independent hESC-CMs differentiation attempts resulted in variance in the percentages of cTNT-positive cells on day 15 of differentiation, ranging from about 15% to 60%, as measured by FACS (Table S2, column 1). After applying a 24 h treatment, the percentages of cTNT-positive cells in the population grew significantly in each attempt (Table S2, columns 2–3). Interestingly, we detected a trend in the fold-change of hESC-CMs percentage after treatment between the different outputs by performing a linear regression analysis (Fig. S1.5). Such a trend suggests there is an association between the yield of differentiation (i.e. %cTNT^+^ before treatment) and the outcome of mBax_499 mRNA treatment (i.e. %cTNT^+^ after treatment). Therefore, we developed a deterministic non-linear model for the prediction of treatment outcome in an attempt to substantiate such correlation by fitting to the experimental data. Finding a relationship between both variables (cTNT-positive percentage of the cell population before and after treatment) can determine whether repetition of mBax_499 mRNA treatment can elevate hESC-CMs enrichment.

By assuming that the population after differentiation is categorized into two groups of nonmyocytes (cTNT^−^) and hESC-CMs (cTNT^+^), the model is based on the principle that a single mBax_499 mRNA treatment is characterized by constant values of nonmyocyte survival rate ($S$) and hESC-CM recovery rate ($yield$) which both depend on the composition of the apoptotic population after treatment. To identify the phenotype of the apoptotic cells in the mBax_499 mRNA treated culture, the cells were co-stained with SIRPa as a cardiac marker and Annexin V as an apoptosis marker, and the cell population was analyzed by FACS. Only 8.5% of the treated apoptotic cells were positive for SIRPa (Fig. 3c, right), after subtracting the basal values which exist due to preparations for analysis (Fig. 3c, left). Therefore, the ratio of hESC-CMs vs. nonmyocytes in the apoptotic cell population was defined as 8.5% vs. 91.5%, respectively. Assuming that this is a fixed ratio for any given treatment, both coefficients $S$ and $yield$ were calculated based on the collected data and determined as the average values of 0.26 ± 0.12 and 0.78 ± 0.19, respectively (see additional information for detailed calculations in Supplementary file). Interestingly, the calculated survival rate (26%) is almost complementary to the previously calculated 80% mRNA translation rate (Fig. 2f), which provides initial validity to our calculations.

In case of repetition of treatment, this model also assumes that each 24 h of treatment is independent of one another and the proliferation of hESC-CMs can be neglected in respect to the proliferation rate of remaining non-differentiated cells. For the non-differentiated cells, ongoing repeated treatment for several days requires the recognition of proliferation of the heterogeneous nonmyocyte population, which can consist of various cell types from undifferentiated to partially differentiated hESCs. Thus, a correction for nonmyocyte survival rate is incorporated as a cell division rate (α) of cell doubling per 24 h treatment, in case more than one treatment is applied. Because proliferation decreases during differentiation progression and the doubling time of undifferentiated hESCs is approximately 24 h^29^, the value of α can range from a minimum of 1 (for non-proliferating cells) to a maximum of 2 (for undifferentiated hESCs). Due to the unknown composition of the nonmyocyte population, we used an estimation of an average value (α = 1.5) in the model.

The model was determined as follows:

$$\begin{array}{rcl}C{M}_{AT} & = & \frac{yiel{d}^{n}\cdot C{M}_{BT}}{((100-C{M}_{BT})\cdot {\alpha }^{n-1}\cdot {S}^{n}+yiel{d}^{n}\cdot C{M}_{BT})}\times 100 \% \\  & = & \frac{{0.78}^{n}\cdot C{M}_{BT}}{((100-C{M}_{BT})\cdot {1.5}^{n-1}\cdot {0.26}^{n}+{0.78}^{n}\cdot C{M}_{BT})}\times 100 \% \end{array}$$

*CM*_*BT*_ - %cTNT^+^ before treatment

*CM*_*AT*_ - %cTNT^+^ after treatment

*n* - Number of repeated treatments

By fitting the data collected from the six independent experiments to the model (Fig. 3d), we substantiate the case for the outcome of mBax_499 mRNA treatment being subjected to a specific nonmyocyte survival rate and hESC-CM recovery rate that were calculated (Chi-square test for goodness of fit, n = 6, χ^2^ = 3.25, *p* > 0.66). This supports the claim that such correlation between the outcome of treatment and the yield of differentiation does exist and suggests that multiple treatments have a beneficial outcome. By performing three consecutive mBax_499 mRNA treatments with 24 h intervals, we improved the outcome of treatment as the cTNT-positive cell population grew by over 4-fold (Fig. 3e). Follow up of cTNT + percentage after every repeated treatment shows precise similarity to the model’s output (Fig. 3f). This provides strong validity for the various assumptions that were made and supports the claim that this model can predict the outcome of repeated treatment; hence it can predict the number of repeated treatments needed to isolate (% cTNT^+^ ≥ 99%) hESC-CMs, given the initial hESC-CM yield after 15 days of differentiation (Table S3).

### MBax_499 reduces safety concerns of hESC-CMs for clinical use

**Figure 4:**
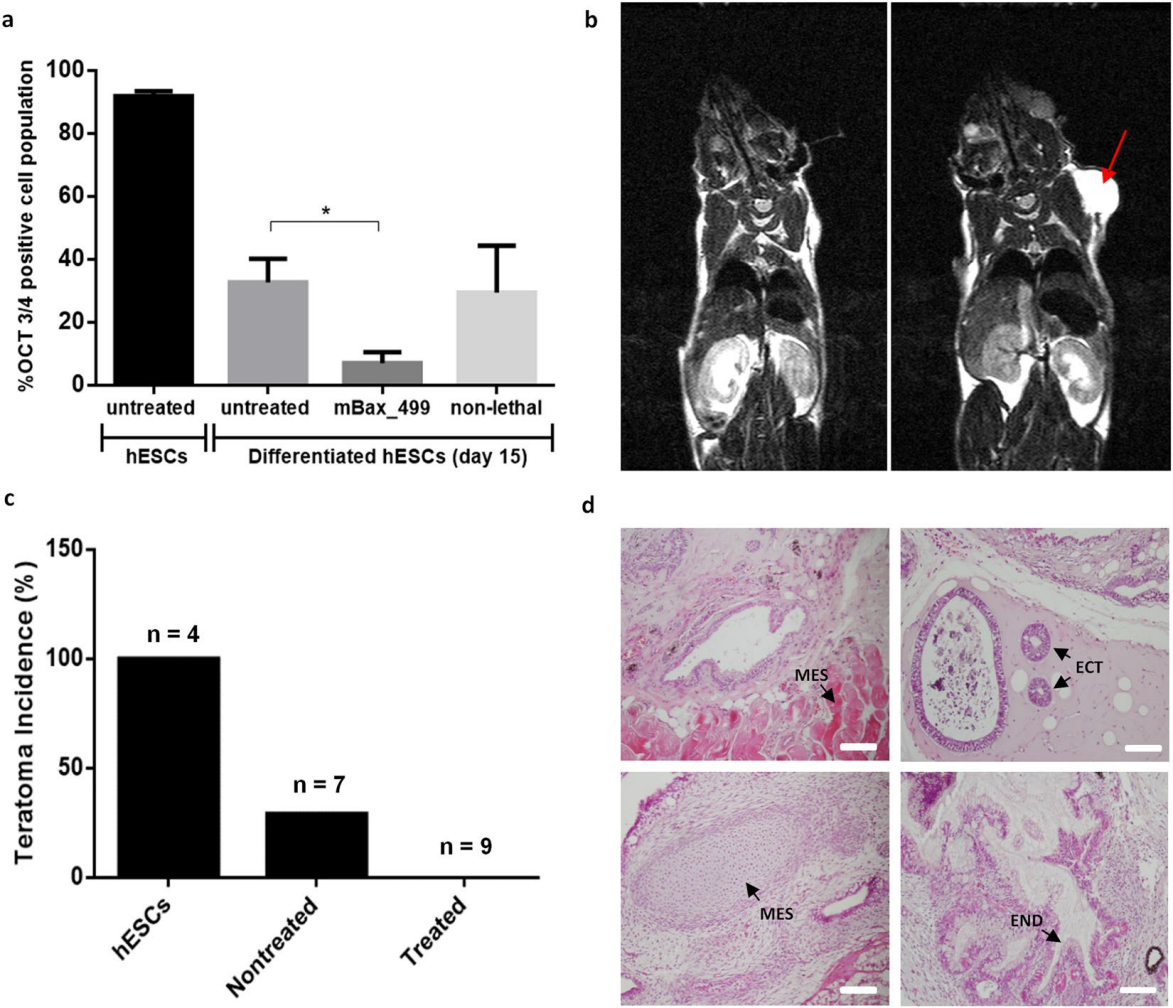
Transplantation of mBax_499 mRNA treated hESC-CMs and testing for teratoma formation. Cardiac differentiated hESCs were treated with mBax_499 mRNA at day 15 for 24 h. (**a**) FACS analysis of cells stained with OCT3/4 for pluripotency (n = 3). *p* value was generated using one-way ANOVA with Tukey’s post-hoc test for multiple comparisons. *p < 0.05. (**b–d**) Teratoma formation was evaluated by injecting 10^6^ cells in matrigel subcutaneously into SCID/NOD mice. (**b**) selected images of MRI body scan of subject without teratoma (left) and subject with teratoma (right - red arrow). (**c**) Bar graph representing the summarized results. (**d**) Selected images of Hematoxylin & Eosin staining of embedded tissues, dissected from mice that developed teratomas, presenting structures from all three germ layers. MES – mesoderm, ECT – ectoderm, END – endoderm. Scale bar: 100 µm.

Next, we investigated whether the enrichment process eliminates the risk of teratoma formation after transplantation. Cardiac differentiated cells (day 15), prior to and after enrichment, were first evaluated for remaining OCT 3/4 positive cells compared to undifferentiated cells. Prior to enrichment, 37% of the cells were positive for the pluripotent marker OCT3/4, while after enrichment, the value was only 7% (compared to the 97% positive cells in undifferentiated hESCs) (Fig. 4a).

These three cell populations were subcutaneously injected between the scapulars of immunocompromised nonobese diabetic severe combined immunodeficient (SCID/NOD) mice. Approximately three months later, magnetic resonance image (MRI) body scan revealed that 4/4 (100%) of the animals injected with undifferentiated hESCs developed teratomas. In the group injected with cardiac differentiated cells (day 15) without enrichment, the rate was 2/7 (29%), while none (0/9) of the mice injected with cardiac cells after treatment with mBax499 construct developed teratomas (Figs 4b,c, S1.6). Histological examination showed that the teratomas generated by hESCs and non-treated cells contained structures characteristic of the three embryonic germ layers (Fig. 4d).

### MBax_499 mRNA treatment does not alter the physiological activity of hESC-CMs

**Figure 5:**
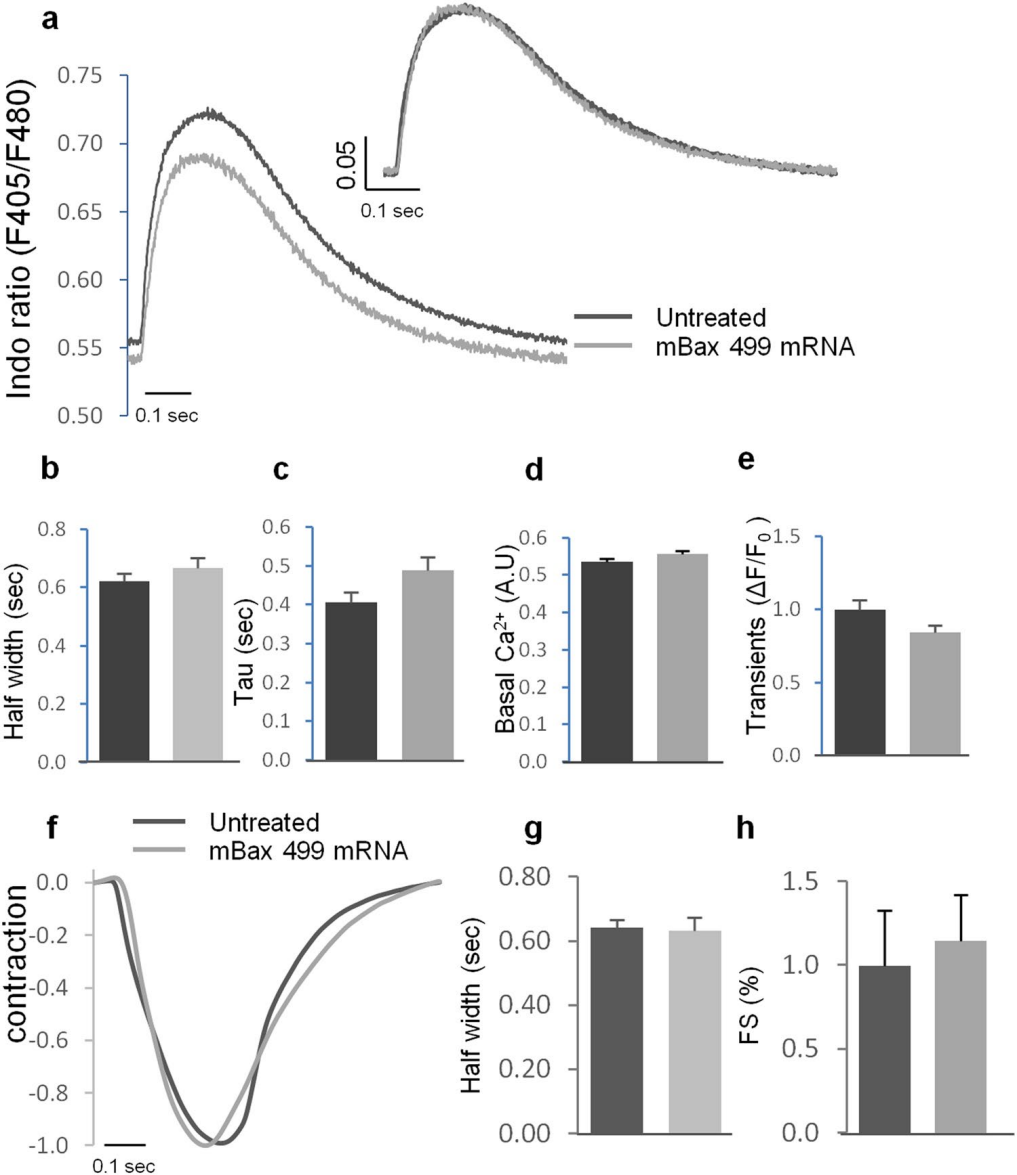
Physiological analysis of hESCs-CMs treated with mBax_499 mRNA. Untreated cells and cells treated with mBax_499 mRNA for 24 h were examined for changes in calcium homeostasis and mechanical contraction. All slides were stimulated with 1 Hz and [Ca^2+^]_i_ transients and contraction signals were recorded and analyzed. (**a**) Representative illustration of average transients from untreated cells (black) or hESC-CMs treated with mBax_499 mRNA (gray). Left sided graph shows average of raw traces and the right graph shows super imposed traces (normalized to peak high). (**b–e**) Parameters measured from calcium transients: (**b**) Half width was calculated as duration from 50% time to [Ca^2+^]_i_ peak to 50% time to calcium decay. (**c**) Tau characterized the speed of calcium reuptake. (**d**) Basal [Ca^2+^]_i_ during relaxation. (**e**) Magnitude of transients represents the % of [Ca^2+^]_i_ alternations during contraction, calculate as ΔF/F_0_ and normalized to untreated cells. (**f–h**) Parameters measured from contraction traces: (**f**) Representative illustration of average super imposed transients of mechanical contraction (normalized to peak high). (**g**) Half width was calculated as duration from 50% time to contraction peak to 50% time to relaxation. (**h**) Fractional shortening (%) characterized the percent change during contraction, normalized to untreated cells. All bar graphs indicate mean (SE). Data was collected from at least 5 different zones obtained from 5–6 independent slides. Parameters were calculated from an average of 15–20 successive transients for each zone. Two-tailed unpaired student’s *t-test* was performed to ensure no statistical differences.

Although hESC-CMs are capable of surviving transfection with mBax_499 mRNA, it is critical to determine whether such treatment has any physiological implications. Thus, calcium and contractility measurements were evaluated in early stage hESCs-CMs (day 15) treated with mBax_499 mRNA in order to compare their functionality with untreated hESC-CMs. All cells showed spontaneous and synchronized (at 0.5 Hz and 1 Hz) intracellular Ca^2+^ ([Ca^2+^]i) transients and clear contraction responses. [Ca^2+^]_i_ transients and super-impose traces during stimulation with 1 Hz revealed similar [Ca^2+^]_i_ kinetics (Fig. 5a). These results demonstrate that treatment with mBax_499 mRNA does not impair [Ca^+2^]_i_ response, as well as mechanical contractility of early stage hESC-CMs, which was revealed by measuring half width duration (Fig. 5b) and tau (Fig. 5c). Additionally, we did not observe any difference in basal [Ca^2+^]i and [Ca^2+^]i transient amplitudes (Fig. 5d,e, respectively). Similar results were obtained using spontaneous beating and during stimulation with 0.5 Hz (data not shown). Overall, our analysis indicates no detectable difference in the contraction parameters of the different hESC-CMs groups for contraction amplitude, half width and fractional shortening, respectively (Fig. 5f–h).

## Discussion

In this work, we describe a novel suicide-based strategy for the efficient elimination of residual undifferentiated hESC from cardiac differentiated cells without affecting the functionality of the cardiac cells. The strategy utilizes the ability of hESCs to rapidly undergo apoptosis by administering a miR-499-responsive mRNA construct that encodes for the constitutively active mutant (S184del) pro-apoptotic BAX variant. While this construct is lethal towards undifferentiated hESCs, it is considered safe in cardiac differentiated cells due to the over-expression of miR-499, which acts as an effective OFF switch of the construct. Expression of mBAX in hESCs culture led to induction of massive apoptosis, as within 24 h nearly 90% of the cells died after cell treatment with 1 pg/cell of the construct (Fig. 2). Such level of lethality and robustness has been further highlighted by the protein translational results measured by the Western Blot analysis, which indicated that a minor amount of mBax units are sufficient for stimulating efficient cell apoptosis and hESC death. The no measurable increase in mBax protein in Western blot assay 24 h after transfection of hESCs is the consequence of rapid cell death. Its translation to a protein and participation in cell killing can be deduced from the following evidences. First, we showed that the construct can undergo translation in mammalian cells to produce mBax protein using a cell free translation kit (Fig. S1.1c) and that synthetic mRNA can undergo cellular uptake and translation in hESCs (Fig. 2e). We also showed that the protein encoded by the construct is responsible for cell death since the cells survived once the transcript was inhibited by miR-499 mimic (Fig. S1.2d). Finally, we showed that the cells died through Bax-mediated apoptosis using multiple assays (Fig. 1c–e).

Given the extreme lethality of the mBax construct, selection of miR-499 as the silencing mechanism, i.e., the OFF switch for suppressing apoptosis mediated by mBax mRNA was appropriate. When the lethal mRNA was placed under the regulation of the cardiac abundant miR-499, it selectively eliminated hESCs and other non-target cells within a heterogeneous population, yet bypassing the majority of early stage hESC-CMs as soon as day 15 of differentiation. This allowed the elimination of residual hESCs and their corresponding tumorigenic potential and the enrichment of hESC-CMs at an early stage of differentiation. In comparison, analysis of the literature reveals that the lactate selection method, commonly used for hESC-CMs selection, is an effective enrichment method only in late-stage cultures^10^. In early stage cultures with the extensive hESC-CM proliferation, the use of lactate as energy source is insufficient for maintaining ATP levels and cell viability due to the high metabolic demands in these cultures. The importance of performing enrichment during the early stage of differentiation has been recently highlighted in a study showing that early stage cardiomyocytes hold the greatest ability to emulate the physiology of the adult myocardium following maturation-related stimulation^17^.

Importantly, the physiological analysis performed on hESC-CMs treated with mBax_499 mRNA showed that the treatment does not impair the contractility and intracellular Ca^2+^ response of early stage hESC-CMs as well their morphology, thus supporting the use of mBax_499 mRNA as a safe method for enrichment and selection at early stage cultures.

By developing a prediction model, which was substantiated by experimental data, we revealed a correlation between the yield of hESC-CM differentiation on day 15 and the extent of enrichment after one or more treatment/s with mBax_499 mRNA. This enabled us to uncover the potential of mBax_499 mRNA construct to completely isolate hESC-CMs (% cTNT^+^ ≥ 99%) and thus remove any of the remaining OCT3/4 positive cells that survive a single treatment. This can be achieved by performing multiple repeated treatments, depending on the initial percentage of hESC-CMs in the cell population. The transfection with the construct had no effect on the differentiation to cardiomyocytes and the functionality of the hESC-CMs and it is expected that multiple transfections would not have an effect on these characteristics, as has previously shown^30^. We will verify such expectation in future studies. In addition, for simplification of the purification strategy, we plan to pursue strategies to reduce the number of repetitions. Such strategies include improving intracellular mRNA delivery and greater translational efficiency (larger than the 80% that was achieved in this work) or adapting a cardiac differentiation protocol that provides high hESC-CM yield. An additional strategy is to directly increase hESC-CM recovery by considering insufficient mRNA:miRNA sensitivity, which results in several mBax_499 mRNA transcripts avoiding the miR-499 silencing mechanism. An attempt to resolve this issue was by inserting an additional miR-499 target in the 5′UTR, although such attempt did not increase sensitivity. A different approach is based on the sequence-based computational tool ViennaRNA package, which can be exploited to improve site accessibility by reducing the energetic cost of freeing base-pairing interactions within the miRNA target region^31^. Additionally, the impact of site abundance on available miRNAs within the cell could very well contribute to undesired translation and can be investigated as well^32^. While the remaining percentage of OCT3/4 positive cells in the treated population remains a safety concern, we believe the following approaches for improvement will assure this method could be applied in practice.

Due to the distinct expression patterns of miRNAs in various cell populations (i.e. liver-specific miR-122 and endothelial-specific miR-126), this versatile system, which can be modulated by a simple adjustment to any desired miRNA is potentially relevant to other hESC-derived populations. Moreover, the use of synthetic mRNA does not significantly disrupt the normal activity of endogenous miRNAs due to its low stability and rapid degradation^33^. While several studies demonstrated that chemical modifications of synthetic mRNAs are necessary for the reduction of immunogenicity^34–36^, the successful administration of unmodified eGFP mRNA (~1 Kb) throughout this work provides strong evidence that short unmodified mRNA does not provoke innate immune and toxic response in hESCs before and after differentiation. While only undifferentiated hESCs are known to have attenuated antiviral response^37,38^, these results suggest that mRNA modifications for short mRNAs (of up to 1Kb) can be avoided when working with early stage hESC-derived populations as well. Whether this is also the case with mRNAs over 1 Kb remains to be investigated.

Overall, this study sheds light on the potential use of such miRNA-responsive lethal mRNA for the safe removal of selected cell populations and can have important implications for cell based therapy strategies as well as suicide gene therapy for cancer treatment.

## Methods

### Cell culture

H9 hESCs (WA09) were obtained from WiCell Research Institute (Madison, WI). The hESCs were cultured and expanded in their undifferentiated state on feeder-free Matrigel-coated plates (BD Biosciences) and maintained in NutriStem hESC XF medium (Biological Industries). Cells were cultured under standard conditions at 37 °C and 5% CO_2_, in a humidified incubator. Cultures were passaged every 3–4 days by incubation in Versene (GIBCO) for 4 min at 37 °C, dissociated and then seeded at ratios between 1:10 to 1:15 onto new Matrigel-coated plates and maintained in NutriStem hESC XF medium with additional 5 µM ROCK inhibitor (Y-27632) for the first 24 h.

### Cardiac monolayer differentiation by small molecule supplementation

Cardiac-directed differentiation was performed via modulation of the regulatory elements of Wnt signaling using chemical inhibitors, according to a previously published protocol^3^ with slight modifications. Briefly, undifferentiated hESCs dissociated into single cells with StemPro Accutase were seeded at a density of 8 × 10^5^ cells/well on 12-well plates with 1.5-fold Matrigel. The seeded cells were cultured in NutriStem supplemented with 5 µM ROCK inhibitor (Y-27632) for 24 h (day −5). Cells were then cultured in NutriStem medium, which was replaced daily. After 5 days, the cells were supplemented with GSK3 inhibitor (CHIR99021) in RPMI supplemented with B27 without insulin (B27-insulin) for 24 h followed by new RPMI/B27-insulin medium. After two days, 5 µM of Wnt production-2 inhibitor (IWP2) was added for an additional two days and was removed by changing the medium to RPMI/B27-insulin for an additional two days. Finally, cells were maintained in RPMI/B27 medium that was changed every other day. Spontaneous cell contraction first appeared on days 8–10 of differentiation.

### Imaging flow cytometry

Samples were dissociated with TrypLE/Collagenase and were fixed using the Foxp3/Transcription Factor Staining Buffer Set (eBioscience, San Diego, CA) according to manufacturer’s instructions. ImageStream^x^ (Amnis, Seattle, WA) was used for cell imaging and acquisition of immuno-staining. Analysis was performed using the IDEAS 6.0 software. Cells were stained with relevant antibodies suspended in FACS buffer (PBS with 2% FBS, v/v). Antibodies: primary anti-cTNT (ab8295, 1:100, Abcam) along with secondary donkey anti-mouse Alexa 488 antibody (715-545-151, 1:250, Jackson) as well as conjugated APC anti CD172 a/b (SIRPa, 323810, 1:100, Biolegend) and AF647 anti OCT3/4 (sc-5279, 1:100, Santa Cruz). 7 Aminoactinomycin D (7-AAD) (Sigma, A9400) was used for apoptosis assay. For cellular uptake, GFP mRNA was labeled with Cy5 using the *Label* IT Nucleic Acid Labeling Kit (Mirus, Madison, WI) according to the manufacturer’s instructions.

### Fluorescence-activated cell sorting (FACS)

Samples were dissociated with TrypLE/Collagenase, stained with SIRPa surface marker and sorted using FACS Aria II (BD Biosciences, San Jose, CA). Sorted cells were used immediately for downstream assays.

### Confocal Imaging

Cells were fixed in 4% (v/v) formaldehyde for 7 min, washed twice in PBS containing 1% BSA, and permeabilized with 0.1% (v/v) Triton-X 100 for 1 h. The samples were incubated overnight in ice with primary antibody against anti-sarcomeric α-actinin (Clone EA-53, 1:300, Sigma) followed by 45 min incubation in RT with Alexa 488-conjugated donkey anti-mouse antibody (715-545-151, 1:250, Jackson) and Alexa-Fluor 546-conjugated phalloidin (A22283, 1:250, Life Technologies) which was used for staining F-actin. Before analysis the samples were washed twice with PBS containing 1% BSA and NucBlue (R37605, Invitrogen) was added for nuclei detection. Imaging was performed with Nikon C1si laser scanning confocal microscope (LSCM). Sarcomeric striation of single cells was quantified with the ImageJ software using the OrientationJ plugin and the distribution application [http://big.www.epfl.ch/demo/orientation/]. OrientationJ calculates the distribution of orientations of fibers in an image by evaluating the structure tensor^39^. The extent of striation was determined as the percentage of actinin fibers that are aligned within 5° of the direction of the alignment which was determined as the mode of the angle distribution (Fig. S1.3).

### Gene expression

Total RNA was isolated using the miRNeasy Micro kit (Qiagen, Germany); concentration and purity were measured on a NanoDrop 1000 spectrophotometer (ThermoFisher Scientific). For miRNA, each sample was reverse transcribed into cDNA using the Taqman MicroRNA Reverse Transcription Kit (Applied Biosystems, Foster City, CA) and for mRNA, each sample was reverse transcribed into cDNA using the high capacity cDNA reverse transcription kit (Applied Biosystems). For gene expression analysis by qRT-PCR, analysis of mRNA and miRNA expression levels was performed using TaqMan gene expression assays and Taqman Fast Advanced Master Mix (Applied Biosystems), respectively. Reactions were run on a StepOnePlus Applied detection system (Applied Biosystems) and reaction conditions included 40 cycles of 95 °C for 10 s, followed by 60 °C for 30 s. Relative gene expression of target gene was calculated by delta delta Ct method using ACTB or GAPDH housekeeping genes for mRNA analysis and U6 for miRNA analysis.

### Western Blot analysis of Bax protein levels

Western blotting was used to evaluate the extent of Bax protein level following mBax mRNA transfection. At the end of each experiment, the cells were lysed in ice-cold radioimmunoprecipitation (RIPA) buffer (Cell Signaling) supplemented with 1 mM phenylmethylsulfonyl fluoride (PMSF). Total protein quantification was performed using micro BCA protein assay (Pierce Biotechnology). Proteins (20 µg/lane) were separated on a mini-PROTEAN TGX gel (Bio-Rad, Hercules, CA), and then transferred to nitrocellulose membranes (Bio-Rad). After blocking, the membranes were incubated with anti-Bax (ab32503, 1:1000, Abcam). Proteins were visualized using anti-rabbit HRP-conjugated secondary antibodies (Pierce Biotechnology), and standard enhanced chemiluminescence (ECL) procedure (Biological Industries). The signal was detected using ImageQuant LAS4000 image analyzer (GE Healthcare, Pittsburg, PA). Densitometric analysis was carried out using imageJ software (U. S. National Institutes of Health, Bethesda, MD, http://imagej.nih.gov/ij/). Band intensity was normalized to GAPDH.

### Caspase 3 activity assay

Cells were analyzed by Fluorimetric Caspase 3 Assay kit (ab39383, Abcam) using the Synergy Mx microplate reader (400 nm/505 nm). The kit is based on detection of cleavage of DEVD-AFC which is a substrate of Caspase 3 and Caspase 7. Upon cleavage, free AFC emits fluorescence, which is quantified and correlated to Caspase 3 activity in the sample.

### Cell survival rate assay

By correlating between reduction-linked intensity to the amount of remaining living cells, survival rates were assessed using the PrestoBlue cell viability assay (Life Technologies) which is based on the live cell’s ability to reduce resazurin (non-fluorescent) to resorufin (fluorescent). This was held under the assumption that during the 24-h span before analysis, reducing power per cell does not change significantly. This assumption was verified by comparing data with DNA content quantification of each sample using the Hoechst 33258 assay. PrestoBlue working solution was prepared by dilution of PrestoBlue reagent 1:10 in cell culture medium. 4 and 24 h post transfection, PrestoBlue working solution was added to the cells for 1 h at 37 °C and 5% CO_2_. Fluorescent intensity was measured using a microplate reader (Synergy Mx, Bio Tek Instruments, Winooski, VT; 560 nm/590 nm). The percentage of cell survival was obtained after normalizing the data to untreated cells. For DNA content quantification, the dissociated cell pellet was resuspended in 0.02% (w/v) sodium dodecyl sulfate in saline-sodium citrate, pH 7.0 (150 mM NaCl and 15 mM sodium citrate) and incubated for 1 h at 37 °C for cell lysis. Then, Hoechst 33258 solution (2 mg/mL) was added, and the mixture was incubated for 10 min at 37 C. Fluorescence was read using a microplate reader (352/461 nm). The cell number was determined using a standard curve.

### Teratoma formation in mice

10^6^ treated hESC-CMs re-suspended in Matrigel (354234, BD Biosciences) were injected subcutaneously between the scapulars of immunocompromised NOD-SCID mice that were purchased from Envigo (Israel). Untreated hESC-CMs as well as undifferentiated hESCs were injected as well, for reference. Between 9–11 weeks after injection, incidences of teratomas were identified by MRI body scan and evaluated by H&E staining. MRI was performed using the M7 1-Tesla compact ICON system (Aspect Imaging, M7, Israel), equipped with a set of application-specific radiofrequency (RF) 80-mm mouse body coils. For *in vivo* imaging, animals were maintained in an anesthetized state with 1.5% isoflurane in O_2_ and placed on a specially designed heated bed. MRI acquisition parameters included fast spin echo with a repetition time of 2,500 ms and echo time of 74 ms. Fifteen axial slices of 0.25 mm with a gap of 0.1 and a matrix of 256 × 256, field of view of 40 mm and acquisition time of 7.4 min. were collected. Dissected tissues were fixed in 4% (v/v) PBS-buffered formalin, gradually dehydrated in alcohol solutions (70–100%), embedded in paraffin and sectioned into 5 µm thick slices. Serial cross-sections were stained with hematoxylin-eosin (H&E) to detect structures that resemble all three germ layers.

### Measurement of intracellular Ca2+ and cardiomyocyte contractility

Intracellular calcium [Ca^2+^]_i_ signals and mechanical contraction of the hESC-CMs were measured with the Hyperswitch dual excitation and dual emission photometry system and the MyoCam-S fast digital dimensioning video camera, respectively (IonOptix, MA, USA). hESC-CMs were grown on slides coated with Matrigel. Fifteen days after differentiation, cells were incubated for 10 min at 37 °C with medium containing 5 µM Indo-1AM (Molecular Probes) and Pluronic F-127 (Life Technologies) at a dilution of 1:1, followed by a 10 min wash to remove excess dye. Slides were transferred to a perfusion chamber mounted on the stage of an inverted microscope and perfused with HEPES-buffered Tyrode’s solution (TB) containing (in mmole/L) NaCl 140, KCl 5.4, MgCl_2_ 1, sodium pyruvate 2, CaCl_2_ 1, HEPES 10 and Glucose 10 (pH 7.4) warmed to 37 °C. [Ca^2+^]_i_ transients were measured by the F405/F480 ratio of Indo-1AM fluorescence. Contraction of hESC-CMs was assessed using a video based edge detection system to continuously measure the movements of a selected area on the slide. Both signals were recorded during spontaneous beatings and following stimulation at 0.5 Hz and 1.0 Hz using a field stimulator (Myopacer; IonOptix) through two platinum electrodes placed on the sides of the perfusion chamber. Data were analyzed using IonWizard data acquisition system (IonOptix). Half width duration was measured from 50% raise to 50% decline of the [Ca^+2^]_i_ or contraction transients.

### Statistical analysis

Statistical Analysis was performed with GraphPad Prism version 6.05 for Windows (GraphPad Software, San Diego, CA). All variable were expressed as mean (±SD). Results were analyzed either by one-way ANOVA with Tukey’s post-hoc test for multiple comparisons or by two-tailed unpaired student’s *t-test*. p < 0.05 was considered statistically significant. Chi-square test was used for analyzing goodness of fit and linear regression was used for trend detection.

### Study approval

The animal study was reviewed and approved by the Institutional Animal Care and Use Committee (IACUC) of the Ben-Gurion University committee (Authorization number: IL-04-06-2014) and the experiment was performed in accordance with relevant guidelines and regulations.

## Supplementary information

Additional Information

Live imaging of hESCs after transfection with mBax_499 mRNA

## Data Availability

The data generated and/or analyzed during this study are either included in the supplementary information files or available from the corresponding author on reasonable request.
