# Supplementary material for: MiR-499 Responsive Lethal Construct for Removal of Human Embryonic Stem Cells after Cardiac Differentiation
Source: Sci Rep. 2019 Oct 10;9:14490. doi: 10.1038/s41598-019-50899-2 (PMC6787023; doi:10.1038/s41598-019-50899-2)
Supplement: Supplementary file 1 — Additional Information [file 41598_2019_50899_MOESM1_ESM.pdf]

S1 - Additional Information

MiR-499 Responsive Lethal Construct for Removal of Human Embryonic Stem Cells after Cardiac Differentiation

Edan Elovic[1], Sharon Etzion[2], Smadar Cohen[1,2,3, *]

[1]The Avram and Stella Goldstein-Goren Department of Biotechnology Engineering,

[2]Regenerative Medicine and Stem Cell (RMSC) Research Center,

[3]The Ilse Katz Institute for Nanoscale Science and Technology

Ben-Gurion University of the Negev, Beer-Sheva 84105, Israel.

Materials

Matrigel-coated plates were from BD Biosciences (San Jose, CA). ROCK inhibitor (Y-27632) was from R&D Systems, Minneapolis, MN; CHIR99021 and IWP2 were purchased from Tocris, United Kingdom; and collagenase type II (95 U/mL) was from Worthington, Lakewood, NJ. Cell culture reagents: NutriStem hESC XF Culture Media, Dulbecco's modified Eagle's medium (DMEM), L-glutamine and heat-inactivated Fetal Bovine Serum (FBS) were from Biological Industries (Kibbutz Beit-Haemek, Israel). B27 supplemented with and without insulin Roswell Park Memorial Institute (RPMI) 1640 medium, StemPro Accutase Cell Dissociation Reagent, TrypLE and the non-enzymatic cell dissociation reagent Versene were from GIBCO (Gaithersburg, MD). The transfection reagent StemFect was purchased from Stemgent. All reagents were of analytical grade.

mRNA Synthesis

Synthetic mRNA was produced through *in vitro* transcription (IVT) using the viral T7 RNA polymerase and a DNA template.

Construction of IVT DNA Templates

The oligonucleotide sequences used in the construction of IVT templates were designed using the Benchling software. Each sequence includes the 5' UTR, initiation of translation region, coding sequence and a customized 3' UTR of the gene of interest. All oligos were synthesized by Bio Basic (Amherst, NY) and were received on a pUC57 or pUCIDT backbone plasmid. Non-Lethal mRNA was synthesized from pEGFP-N1 plasmid kindly provided by Prof. Ziv Reich from the Weizmann Institute, Israel. Excision of the DNA templates from the backbone and the insertion of a T7 promoter to the 5' terminal was established by PCR reactions using the HiFi Hotstart (KAPA Biosystems, Woburn, MA), per manufacturer's instructions. The pre-designed T7 tailed forward primers and reverse primers were purchased from Synthezza IDT (Jerusalem, Israel) and are shown in Table S1. All the PCR products were purified with GeneJET PCR purification kit (Thermo Fisher Scientific, Carlsbad, CA) before further processing.

In Vitro Transcription of mRNA

RNA was synthesized with the HiScribe T7 ARCA mRNA kit with tailing (New England Biolabs, Ipswich, MA), with 1µg of purified tail PCR product as a template in each 20 µL reaction. A blend of 1mM guanosine triphosphate, 1.25mM adenosine triphosphate, 1.25mM

cytidine triphosphate and 1.25mM uridine triphosphate in addition to 4mM 3′-0-Me-m^7G(5′)ppp(5′)G ARCA cap analog were used in each reaction. The reactions were incubated for 4 h at 37^0C followed by DNase treatment for 15 min. The reaction was concluded with Poly(A) tailing as directed by the manufacturer. RNA was purified with Ambion MEGAclear spin columns, quantified by NanoDrop 1000 spectrophotometer (ThermoFisher Scientific) and evaluated by gel electrophoresis as described below.

RNA Transfection

RNA transfections were carried out with the transfection reagent Stemfect to deliver 1 pg of mRNA per cell. For a 24 well-plate transfection experiment containing 1.5^x10^5 cells/well, 150 ng of RNA and 1 µL reagent (per well) were first diluted in 12.5 µl stemfect transfection buffer, separately. Then, these components were pooled together and incubated at RT for 15 min before being added to the wells dropwise (25 µL of mRNA transfection complex per well) using a pipette. The plate was rocked gently and incubated at 37^oC and 5% CO_2. For a 12 well-plate transfection experiment containing 4.5^x10^5 cells/well, 450 ng of RNA and 2 µL reagent (per well) were first diluted in 25 µL stemfect transfection buffer, separately. RNA transfections were performed in NutriStem hESC XF medium for undifferentiated hESCs and in DMEM with 2% B27 supplement and 1% L-glutamine for differentiated cells.

Determining the nonmyocyte survival rate and hESC-CM recovery rate coefficients for the prediction model of mBax_499 treatment outcome

Let CM_{BT} be the percentage of hESC-CMs before treatment (based on $cTNT^+$ cell population) or in other words, the absolute number of hESC-CMs from a sample of 100 cells. Equivalently, the number of nonmyocytes in that sample would be (100 - CM_{BT}). Let % A be the percentage of the population that underwent apoptosis, which consist of 8.5% hESC-CMs (Figure 3d). Thus, the percentage of hESC-CMs after treatment (CM_{AT}) can be calculated as:

$$CM_{AT} = \frac{CM_{BT} - 0.085 \cdot \%A}{100 - \%A} \times 100\%$$

The equation above can be reorganized in order to calculate the percentage of cells that underwent apoptosis, given both CM_{AT} and CM_{BT} from each experiment:

$$\%A = \frac{CM_{AT} - CM_{BT}}{\frac{CM_{AT}}{100} - 0.085}$$

After determining %A, the nonmyocyte survival rate (S) and can be calculated through the following:

$$S = \frac{(100 - CM_{BT}) - 0.915 \cdot \%A}{(100 - CM_{BT})}$$

Similarly, the hESC-CMs recovery rate (i.e. yield) during treatment can be calculated as well:

$$yield = \frac{CM_{BT} - 0.085 \cdot \%A}{CM_{BT}}$$

The calculated *S* and *yield* from six independent experiments are listed in table S2.

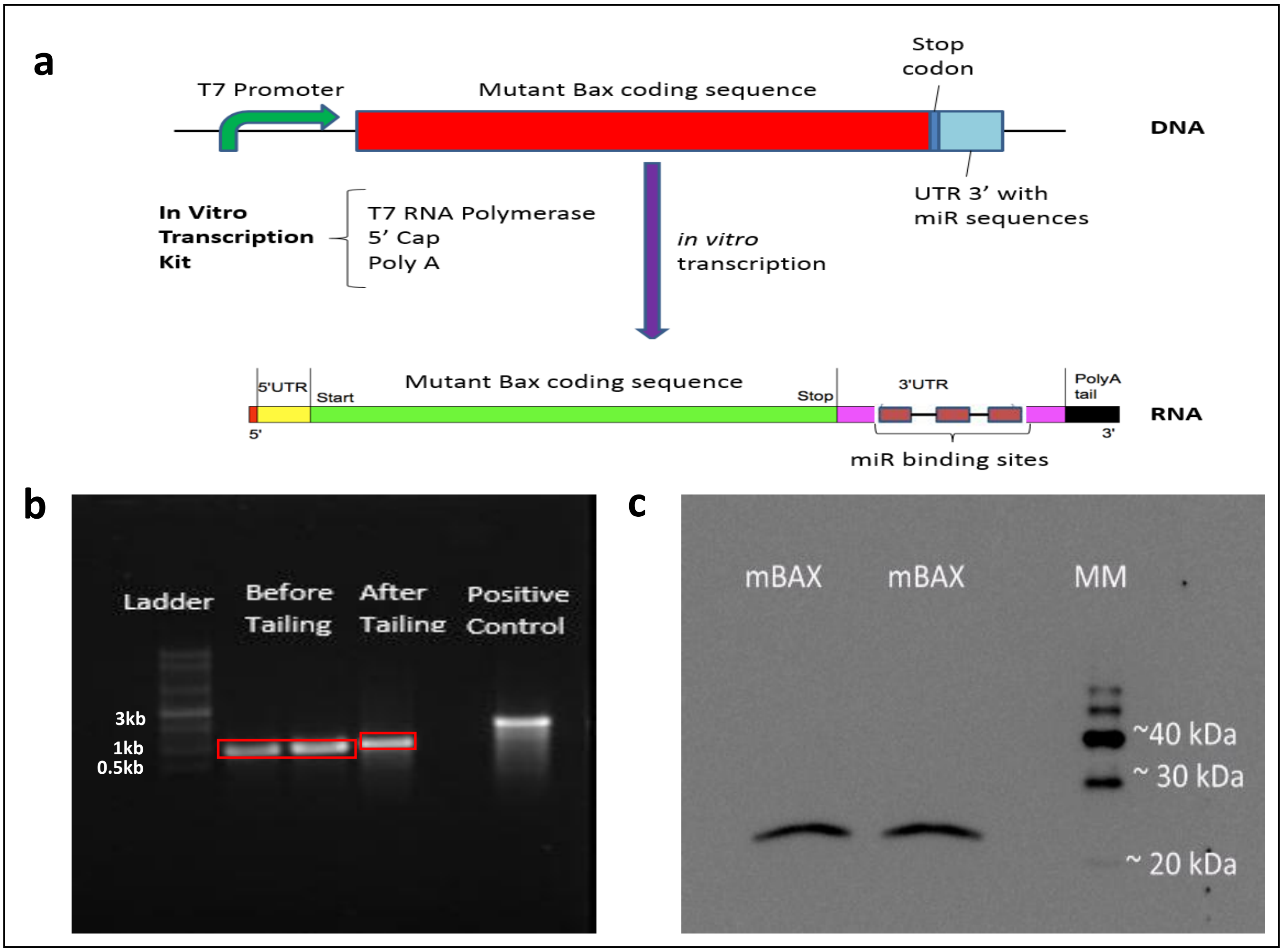

Fig. S1.1 Design and validation of lethal mRNA construct. **a** General scheme of *in vitro* transcription (IVT) of miRNA-responsive mRNA. **b** IVT reaction validation of synthesized mBax mRNA, run on a 1% denaturing agarose gel, stained with ethidium bromide and visualized by UV fluorescence. CLuc mRNA as a positive control and ssRNA ladder by New England Biolabs were used. **c** 1-step human coupled DNA IVT kit (ThermoFisher) was used for cell-free translation of mBax mRNA and the following product (21 kDa) was verified through Western blot**.**

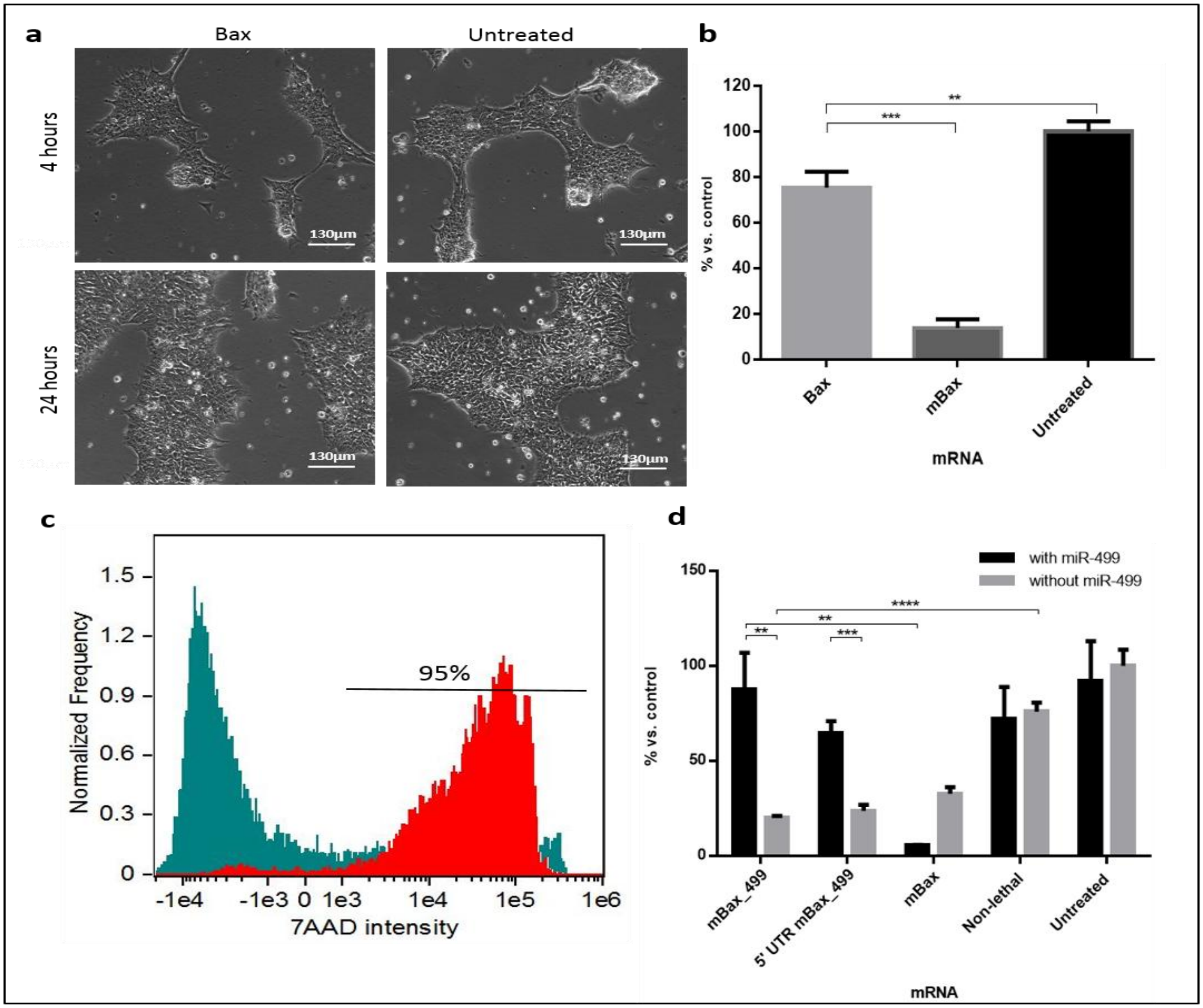

Fig. S1.2 Durability of hESCs to various synthesized mRNAs. **a** Brightfield images of untreated hESCs as well as treated with Bax mRNA for 4-24 h. **b** Viability of hESC treated with Bax and mBax mRNAs for 24 h compared to untreated cells, by Presto blue viability assay (n=3). **c** hESCs, treated with mBax_499 mRNA (red) and non-lethal mRNA (green) for 24 h, after staining with 7AAD and analysis with ImageStream^{X} flow cytometer. **d** hESCs treated with mature miR-499-5p mimic (mirVana miRNA mimic, Ambion) for 5 h prior to treatment with various synthesized mRNAs. All data presented are normalized to untreated group (control), n=3. Presto blue viability assay was performed 24 h after treatment. *5' UTR mBax_499* represents mBax_499 mRNA with an additional miR-499 target at the 5' UTR. All bar graphs indicate mean (SD). *p* values were generated by using one-way ANOVA with Tukey's post-hoc test for multiple comparisons. $^{**}p < 0.01$, $^{***}p < 0.001$, $^{****}p < 0.0001$.

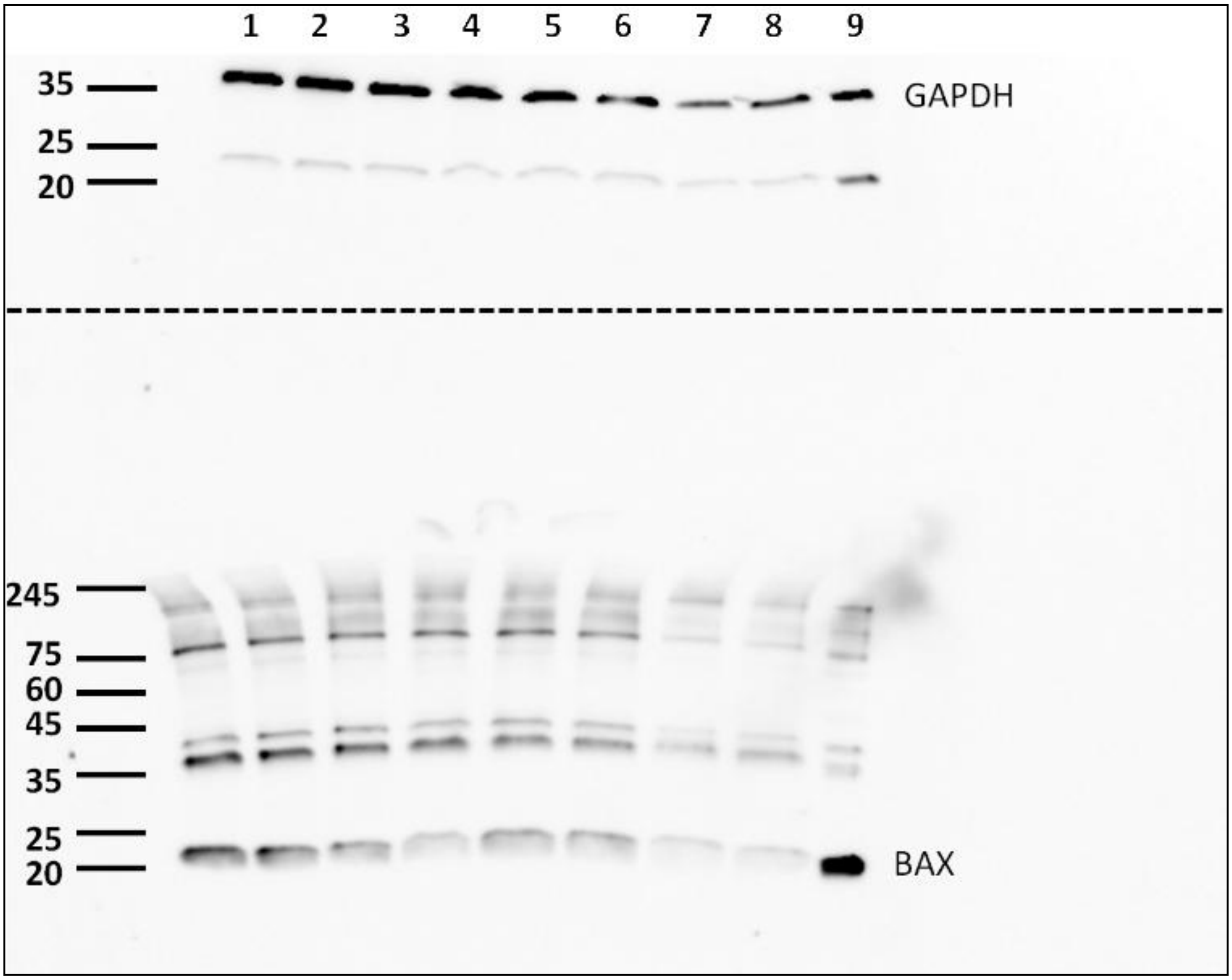

Fig. S1.3 Full-length blots with low exposure corresponding to Figure 1f. Both blots are derived from the same experiment and were processed in parallel. Lanes 1:2 – untreated cells, lanes 3:4 - eGFP mRNA, lanes 5:6 – Bax mRNA, lanes 7:8 – mBax mRNA, Lane 7 – mBax protein produced by cell-free translation of mBax mRNA. The protein marker PM2600 (SMOBIO) was used to identify bands that belong to BAX (~21 kDa) and GAPDH (~36 kDa) proteins.

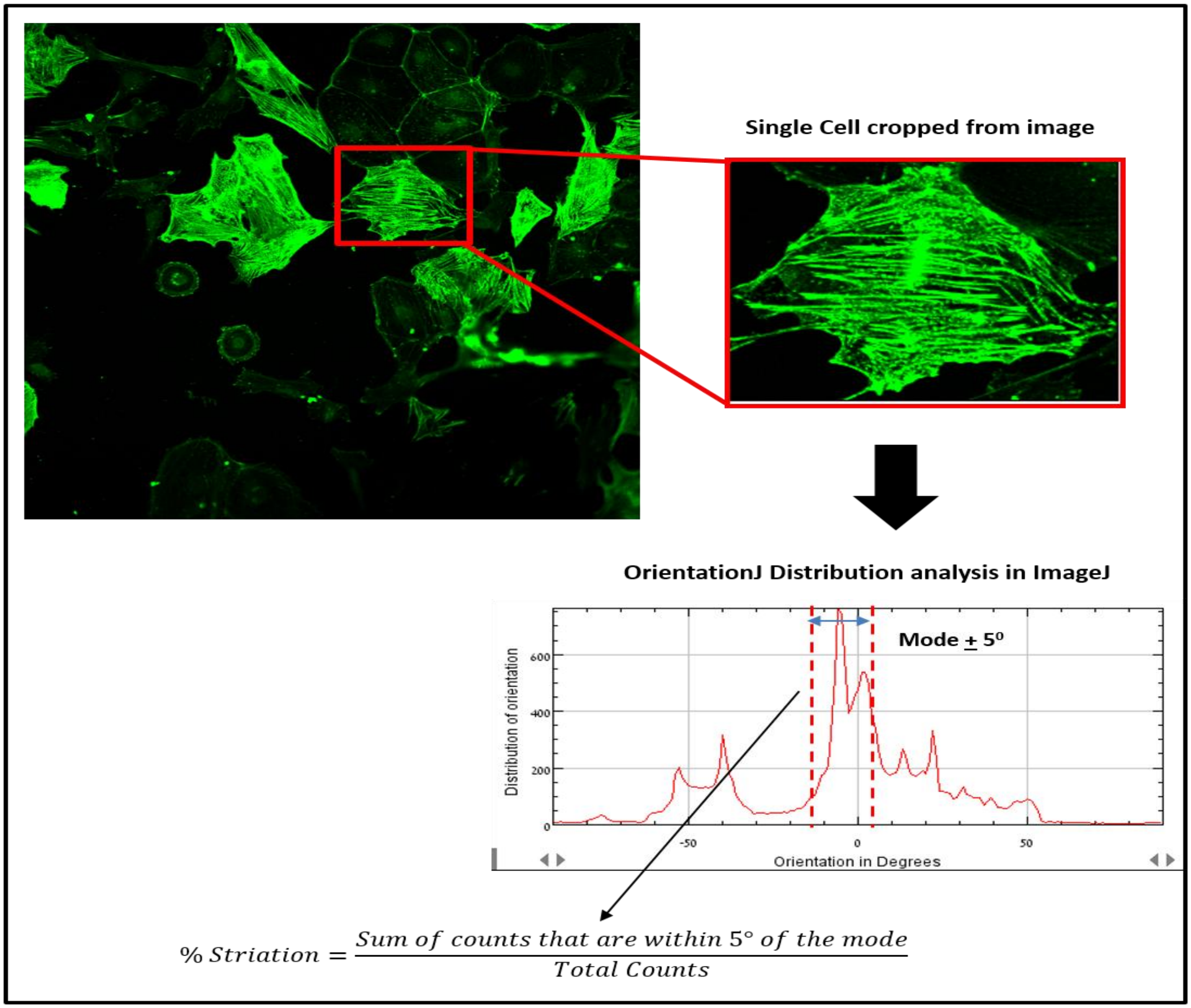

Fig. S1.4 Detailed method for the quantification of the extent of striation of hESC-CMs stained with sarcomeric α-actinin using Image J software and the Orientation J Distribution plugin.

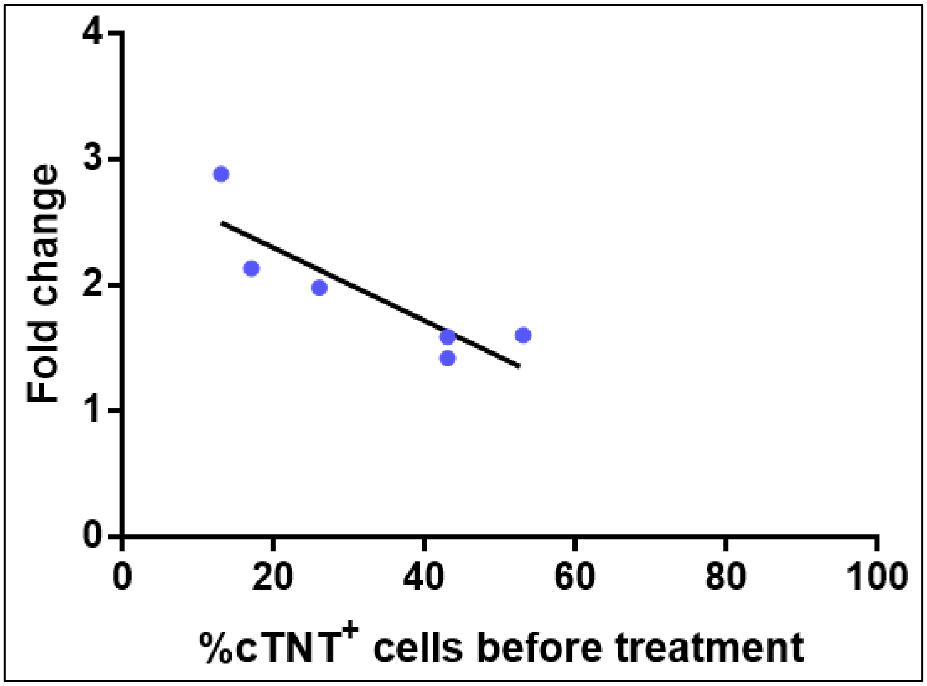

Fig. S1.5 Linear regression of cTNT-positive percentage of cells before mBax_499 mRNA treatment vs. fold change of cTNT-positive percentage of cells after treatment. Blue dots represent experimental results (mean fold-change). $r^2 = 0.76$ and $p = 0.0236$.

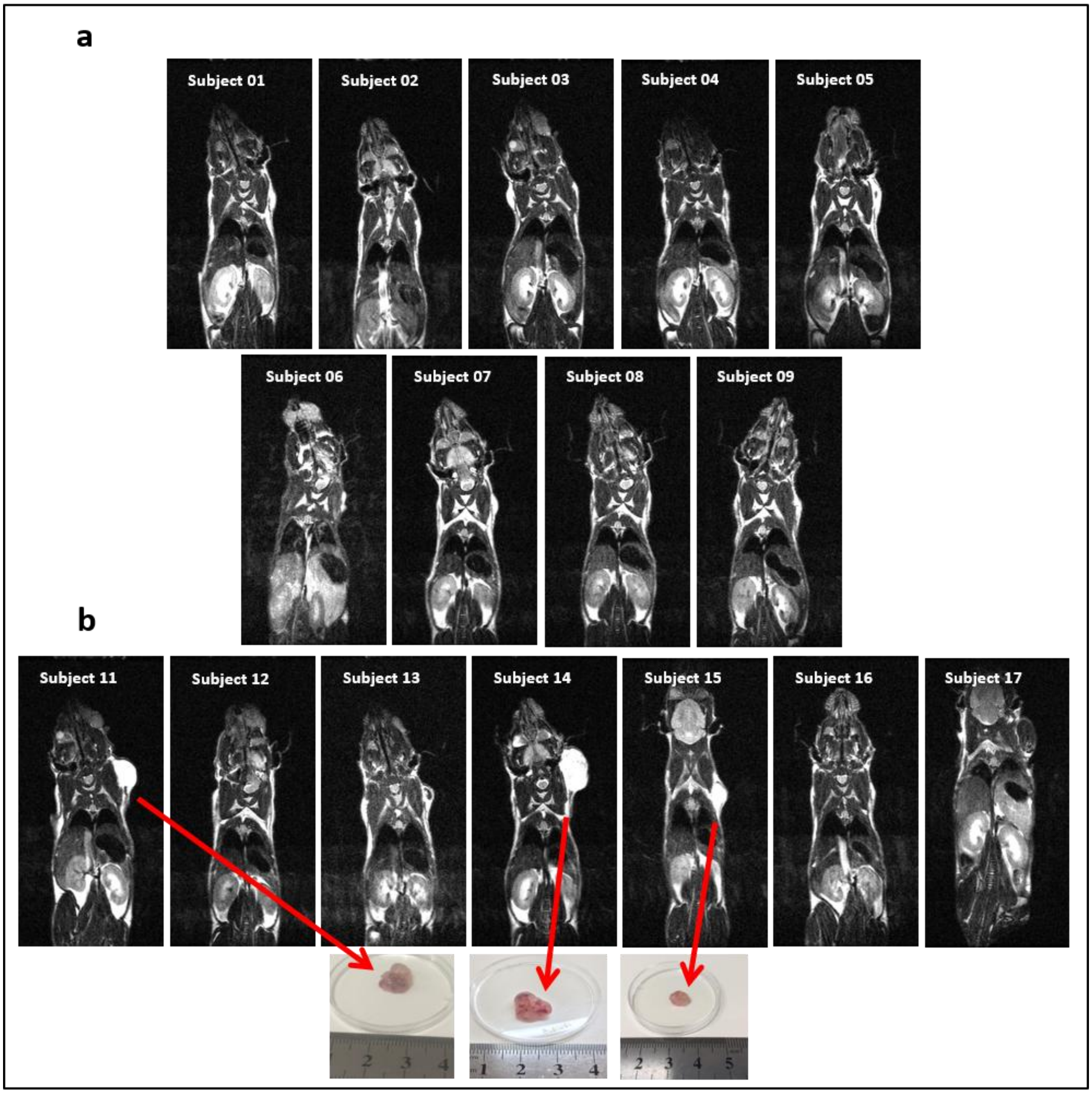

Fig. S1.6 MR Images of mice 9-11 weeks after injection of differentiated hESCs. **a** Mice injected with differentiated hESCs treated with mBax_499, n=9. **b** Mice injected with differentiated hESCs that were not treated with mBax_499 mRNA and the corresponding images of the dissected tumors identified in MRI, n=7.

Table S1: Primers used for the IVT DNA templates. T7 promoter sequence is marked in bold.

mRNA	Primer	Sequence
mBax_499	Forward	**TTAATACGACTCACTATAGG**ATCACACCGGTCGCCACC
	Reverse	TAGCTAGCATCTCGAGGAGTGTC
eGFP	Forward	**TTAATACGACTCACTATAGG**ATCCACCGGTCGCCACCAT
	Reverse	TCAGGTTCAGGGGGAGGTGTGG
mBax	Forward	**TTAATACGACTCACTATAGG**ATCACACCGGTCGCCACC
	Reverse	TAGCTAGCATCTCGAGGAGTGTC
Bax	Forward	**TTAATACGACTCACTATAGG**ATCACACCGG
	Reverse	TCAGGTTCAGGGGGAGGTGTGG

Table S2: Summary of six independent hESC-CM differentiation attempts and their corresponding coefficients.

Column	1	2	3	4	5	6
**Experiment**	**%cTNT⁺ (day 15) before Treatment**	**%cTNT⁺ after 24 h Treatment**	**P Value**	*%A*	*S*	*yield*
1	13% ± 1.8%	37.5% ± 0.3%	0.0028	84.5%	0.111	0.448
2	43% ± 1.6%	68.5% ± 3.2%	0.0003	42.5%	0.318	0.916
3	26% ± 4%	51.5% ± 1.1%	0.0137	59.3%	0.267	0.806
4	17% ± 0.6%	36.3% ± 2.2%	0.0001	69.4%	0.235	0.653
5	53% ± 3.3%	85% ± 5.2%	0.0008	41.8%	0.186	0.933
6	43% ± 1.7%	61.1% ± 0.9%	<0.0001	34.4%	0.448	0.932
**Average**					**0.261**	**0.781**
**SD**					0.116	0.196

Columns 1, 2: Percentage of $cTNT^+$ in the cell population at day 15 of differentiation before / after a single treatment with mBax_499 mRNA, determined by FACS. Data indicate mean (±SD). Column 3: *p* values of statistical difference between columns 1 and 2 (which were generated by using two-tailed unpaired student's *t-test*). Columns 4-6: Calculated coefficients for the prediction model for each experiment and their corresponding average and standard deviation. %A – percentage of apoptotic population, S – nonmyocyte survival rate, yield – hESC-CM recovery rate.

Table S3: Outcome prediction of repetition of treatment with mBax_499 mRNA vs. initial hESC-CM's percentage at day 15 of differentiation.

n	1	2	3	4	5	6	7	8	9	10
CM_{BT}	CM_{AT}									
10	25.0	39.9	57.0	72.5	84.0	91.3	95.4	97.7	98.8	99.4
15	34.6	51.3	67.8	80.7	89.3	94.3	97.1	98.5	99.3	
20	42.8	59.9	74.9	85.6	92.2	95.9	97.9	98.9	99.5	
25	49.9	66.6	79.9	88.8	94.0	96.9	98.4	99.2		
30	56.2	71.9	83.6	91.1	95.3	97.6	98.8	99.4		
35	61.7	76.3	86.5	92.8	96.2	98.1	99.0			
40	66.6	79.9	88.8	94.1	96.9	98.4	99.2			
45	71.0	83.0	90.7	95.1	97.5	98.7	99.4			
50	75.0	85.7	92.3	96.0	97.9	99.0				
55	78.5	87.9	93.6	96.7	98.3	99.1				
60	81.8	90.0	94.7	97.3	98.6	99.3				
65	84.8	91.7	95.7	97.8	98.9	99.4				
70	87.5	93.3	96.5	98.2	99.1					
75	90.0	94.7	97.3	98.6	99.3					
80	92.3	96.0	97.9	99.0						
85	94.4	97.1	98.5	99.3						

Values in table represent percentage of $cTNT^+$ cells in population and the numbers 1 through 10 in the first row represent the number of repeated treatment. The number in the first row corresponding to each yellow-marked cell represent the amount of repeated treatment needed for the purification of hESC-CMs (% $cTNT^+ \geq 99\%$).

DNA Template Sequences for IVT

>mBax

ATCACACCGGTCGCCACCATGGACGGGTCCGGGGAGCAGCCCAGAGGCGG

GGGGCCCACCAGCTCTGAGCAGATCATGAAGACAGGGGCCCTTTTGCTTC

AGGGTTTCATCCAGGATCGAGCAGGGCGAATGGGGGGGGAGGCACCCGAG

CTGGCCCTGGACCCGGTGCCTCAGGATGCGTCCACCAAGAAGCTGAGCGA

GTGTCTCAAGCGCATCGGGGACGAACTGGACAGTAACATGGAGCTGCAGA

GGATGATTGCCGCCGTGGACACAGACTCCCCCCGAGAGGTCTTTTTCCGA

GTGGCAGCTGACATGTTTTCTGACGGCAACTTCAACTGGGGCCGGGTTGT

CGCCCTTTTCTACTTTGCCAGCAAACTGGTGCTCAAGGCCCTGTGCACCA

AGGTGCCGGAACTGATCAGAACCATCATGGGCTGGACATTGGACTTCCTC

CGGGAGCGGCTGTTGGGCTGGATCCAAGACCAGGGTGGTTGGGACGGCCT

CCTCTCCTACTTTGGGACGCCCACGTGGCAGACCGTGACCATCTTTGTGG

CGGGAGTGCTCACCGCCCTCACCATCTGGAAGAAGATGGGCTGAGGCCCC

CAGCTGCCTTGGACTGTGTTTTTCCTCCATAAATTATGGCATTTTTCTGG

GAGGGGTGGGAATTCTGAACGTCACTACAAATCAATTCTGAACGTCACTA

CAAATTAATTCTGAACGTCACTACAAAGTAATTCTGAACGTCACTACAAA

CGTCGACTCCTTCGGGACACTCCTCGAGATGCTAGCTA

>mBax_499

ATCACACCGGTCGCCACCATGGACGGGTCCGGGGAGCAGCCCAGAGGCGG

GGGGCCCACCAGCTCTGAGCAGATCATGAAGACAGGGGCCCTTTTGCTTC

AGGGTTTCATCCAGGATCGAGCAGGGCGAATGGGGGGGGAGGCACCCGAG

CTGGCCCTGGACCCGGTGCCTCAGGATGCGTCCACCAAGAAGCTGAGCGA

GTGTCTCAAGCGCATCGGGGACGAACTGGACAGTAACATGGAGCTGCAGA

GGATGATTGCCGCCGTGGACACAGACTCCCCCCGAGAGGTCTTTTTCCGA

GTGGCAGCTGACATGTTTTCTGACGGCAACTTCAACTGGGGCCGGGTTGT

CGCCCTTTTCTACTTTGCCAGCAAACTGGTGCTCAAGGCCCTGTGCACCA

AGGTGCCGGAACTGATCAGAACCATCATGGGCTGGACATTGGACTTCCTC

CGGGAGCGGCTGTTGGGCTGGATCCAAGACCAGGGTGGTTGGGACGGCCT

CCTCTCCTACTTTGGGACGCCCACGTGGCAGACCGTGACCATCTTTGTGG

CGGGAGTGCTCACCGCCCTCACCATCTGGAAGAAGATGGGCTGAGGCCCC

CAGCTGCCTTGGACTGTGTTTTTCCTCCATAAATTATGGCATTTTTCTGG

GAGGAAACATCACTGCAAGTCTTAATCGGAAACATCACTGCAAGTCTTAA

TTTGAAACATCACTGCAAGTCTTAAGTCGAAACATCACTGCAAGTCTTAA

CGTCGACTCCTTCGGGACACTCCTCGAGATGCTAGCTA

>**Bax**

TTAATACGACTCACTATAGGATCACACCGGTCGCCACCATGGACGGGTCC

GGGGAGCAGCCCAGAGGCGGGGGGCCCACCAGCTCTGAGCAGATCATGAA

GACAGGGGCCCTTTTGCTTCAGGGTTTCATCCAGGATCGAGCAGGGCGAA

TGGGGGGGGAGGCACCCGAGCTGGCCCTGGACCCGGTGCCTCAGGATGCG

TCCACCAAGAAGCTGAGCGAGTGTCTCAAGCGCATCGGGGACGAACTGGA

CAGTAACATGGAGCTGCAGAGGATGATTGCCGCCGTGGACACAGACTCCC

CCCGAGAGGTCTTTTTCCGAGTGGCAGCTGACATGTTTTCTGACGGCAAC

TTCAACTGGGGCCGGGTTGTCGCCCTTTTCTACTTTGCCAGCAAACTGGT

GCTCAAGGCCCTGTGCACCAAGGTGCCGGAACTGATCAGAACCATCATGG

GCTGGACATTGGACTTCCTCCGGGAGCGGCTGTTGGGCTGGATCCAAGAC

CAGGGTGGTTGGGACGGCCTCCTCTCCTACTTTGGGACGCCCACGTGGCA

GACCGTGACCATCTTTGTGGCGGGAGTGCTCACCGCCTCGCTCACCATCT

GGAAGAAGATGGGCTGAAGCGGCCGCGACTCTAGATCATAATCAGCCATA

CCACATTTGTAGAGGTTTTACTTGCTTTAAAAAACCTCCCACACCTCCCC

CTGAACCTGA
